# Analysis of Overall Survival in High-Grade Glioma Patients Treated With Surgery and Adjuvant Therapy

**DOI:** 10.7759/cureus.88792

**Published:** 2025-07-26

**Authors:** Sakshi Priya Dewangan, Geeta S Narayanan, Kiran Kumar BR

**Affiliations:** 1 Radiation Oncology, Vydehi Institute of Medical Sciences and Research Centre, Bengaluru, IND

**Keywords:** gbm, glioblastoma, overall survival, radiotherapy, rt, temozolomide

## Abstract

Background

High-grade gliomas (HGGs) are brain tumors that are aggressive and grow quickly. Glioblastoma multiforme (GBM) is the most common, severe, and aggressive subtype of these. Maximal safe resection is the standard course of treatment, which is followed by concomitant radiotherapy and adjuvant chemotherapy. It still has a dismal prognosis and is lethal despite contemporary treatments. The objective of the study is to analyze the median overall survival for GBM patients who underwent primary surgery and adjuvant chemoradiation.

Materials and methods

This was a single-institution retrospective study of 72 patients with HGGs from 2017 to 2022. Data regarding patient factors (age, gender), disease factors (grade, histology), treatment factors (surgery, chemotherapy and radiation therapy), and follow-up information were collected from patient records, and survival was calculated.

Results

A total of 72 patients with HGG were analyzed, and the male-to-female ratio was 2:1. Maximum patients had grade IV glioma (55%), and 85% of the patients presented with a performance score of 1 or 2. Radiation dose was 60 Gy, and chemotherapy used was temozolomide. After completion of the treatment, 59% of the patients presented with progression at the median progression-free survival (PFS) of eight months. The median overall survival was 15 months (95% CI). Females constituted 45.0% (n=8) of survivors, suggesting a trend toward better survival in females. Grade III and oligodendroglioma histology were associated with better survival.

Conclusion

Overall survival in GBM patients remains poor despite constant research and studies. Maximum safe resection followed by adjuvant concurrent chemoradiation and adjuvant temozolomide has shown improvement in overall survival.

## Introduction

Primary brain tumors, known as gliomas, develop from glial cells, a subset of non-neuronal cells that give the primary structure and support to the brain [1]. These tumors are categorized into four different classes according to their clinical behavior and histological features. Gliomas in grades I and II are classified as low-grade gliomas, while those in grades III and IV are classified as high-grade gliomas (HGGs) [2]. HGGs are notable brain tumors that are extremely aggressive and develop quickly, making clinical care extremely difficult. Glioblastoma multiforme (GBM) is the most common, aggressive, and malignant subtype of HGGs [3]. GBMs frequently penetrate the surrounding brain parenchyma and are distinguished by their invasive nature and quick development rate [4]. GBMs seldom spread outside of the central nervous system despite their violent nature [5]. Unfortunately, patients with HGGs, particularly GBM, continue to have a dismal prognosis despite advancements in contemporary therapy [6].

HGGs are being treated with a multi-modal strategy that includes adjuvant chemotherapy and concurrent chemoradiotherapy after maximally safe surgical resection [7]. Although the goal of this aggressive treatment approach is to maximize results, the biology of HGGs, which includes their infiltrative nature, molecular heterogeneity, and tendency to resist traditional therapies, often results in poor long-term survival rates and inevitable recurrence [8].

The aim of this study was to evaluate the median overall survival (OS) of GBM patients who received chemoradiotherapy after primary surgery. In order to shed light on the practical effectiveness of the adjuvant treatments now in use and investigate prospective strategies for enhancing survival in this difficult patient population, we examined a cohort of patients who underwent surgical resection followed by radiation therapy and chemotherapy [9]. In order to optimize treatment procedures and direct future research efforts in the management of HGGs, it is imperative to comprehend how adjuvant therapy affects OS [10].

## Materials and methods

The study cohort was carefully selected through a retrospective review of medical records of patients diagnosed with HGGs who underwent surgical resection followed by adjuvant therapy in our institution from January 2017 to December 2022. After obtaining ethical approval from the Institutional Ethics Committee, medical records of the HGG patients were reviewed (VIEC/2024/APP/77). A total of 72 patients were included in this study based on the following predefined criteria: age 18 years or older at the time of diagnosis with a histopathologically confirmed diagnosis of HGG (grade III or IV), as per the WHO classification of central nervous system tumors [11], and must have undergone maximal safe surgical resection, including gross total resection, subtotal resection, or biopsy, followed by adjuvant therapy, which encompassed radiotherapy (RT) with chemotherapy (e.g., temozolomide [TMZ]). Patients less than 18 years old, those who had previously received radiation, and those with distant metastasis were excluded from the study.

All the patients in the study underwent maximal safe resection. Three weeks after the surgery, MRI was performed to assess the residual status and for RT delineation of the tumor volumes. Adjuvant chemoradiation (CTRT) of radiation dose of 60 Gy in 30 fraction was given along with concurrent TMZ 75 mg/m^2^ of body surface area during the days of the radiation. All the patients after the completion of CTRT received adjuvant TMZ 150 mg/m^2^ body surface area for five days in a 28-day cycle for six cycles. Radiation technique was either 3DCRT (three-dimensional conformal radiotherapy) or IMRT (intensity-modulated radiotherapy). After the completion of the treatment, all the patients were followed up every three months. MRI was performed as a follow-up investigation for the tumor response and progression of the disease. The primary objective of the study was to assess the OS and progression-free survival (PFS) and the secondary objective was to compare the OS and PFS with the age, grade, and histology. All the data related to the patient details, treatment, and follow-up details were documented and analyzed.

SPSS software Version 21 (IBM Corp., Armonk, NY) was used for statistical analysis. Categorical variables were expressed as percentages and analyzed using the chi-square test or Fisher’s exact test. Continuous variables were expressed as mean or median and analyzed using the Mann-Whitney test. Demographic factors and clinical characteristics were summarized as percentages for categorical variables and as median for continuous variables.

## Results

Patient characteristics

The present study included a total of 72 patients with HGG, with a median age of 63 years. Complete details of the patients, tumor, and treatment are given in Table 1, with different analysis results. The cohort comprised 25 (34.7%) females and 47 (65.3%) males. The most common histopathological diagnosis was GBM, accounting for 55.5% (n=40) of cases, followed by anaplastic astrocytoma (23.6%, n=17) and anaplastic oligodendroglioma (20.8%, n=15). Based on the WHO grading system, 61% (n=44) of patients had grade IV tumors. Of these, 18 (25%) patients were alive at the time of analysis, while 54 (75%) patients had died.

**Table 1:** Patient, tumor, and treatment characteristics 3DCRT, three-dimensional conformal radiotherapy; IMRT, intensity-modulated radiotherapy; VMAT, volumetric arc radiotherapy

Variables	Number (%)
Gender	
Female	25 (34.7)
Male	47 (65.3)
Performance status	
0	0
1	26 (36.1)
2	35 (48.6)
3	11 (15.3)
Histology	
Oligodendroglioma	15 (20.8)
Astrocytoma	17 (23.6)
Glioblastoma multiforme	40 (55.5)
Tumor grade	
Grade III	28 (38.8)
Grade IV	44 (61.1)
Radiation technique	
3DCRT	25 (34.7)
IMRT	42 (58.3)
VMAT	5 (6.9)
Total	72 (100)

Overall survival

**Figure 1:**
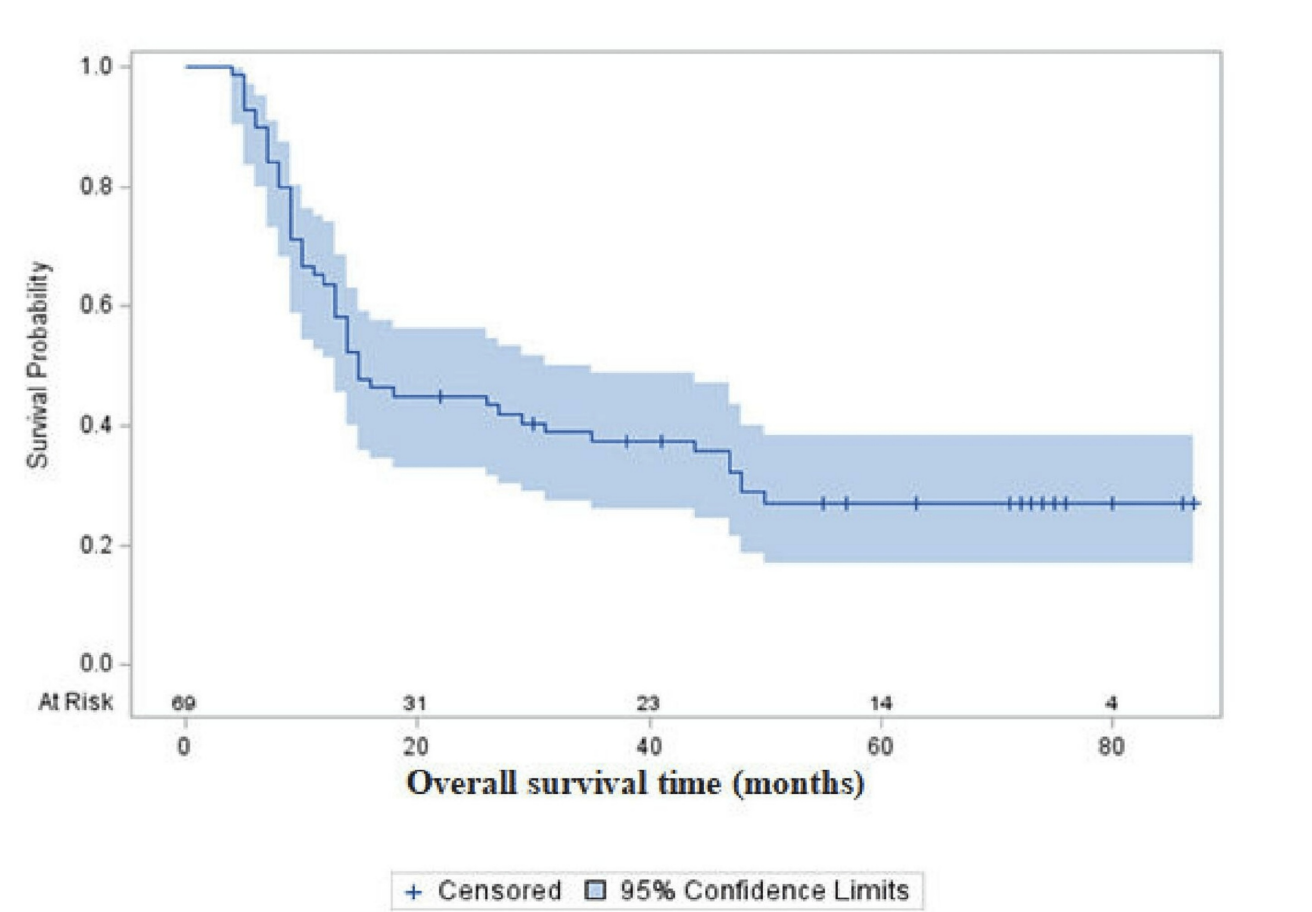
Overall survival of HGG patients who underwent maximal safe resection and adjuvant CTRT and TMZ The Kaplan-Meier curve shows the overall survival of HGG patients who underwent maximal safe resection and adjuvant CTRT and TMZ HGG, high-grade glioma; CTRT, ; TMZ, temozolomide

The median OS was 15 months (95% CI: 13-25) (Figure 1). The OS analysis revealed significant disparities based on tumor grade and histopathological subtype. A total of 18 (25%) patients were alive and were on regular follow-up at the time of the study. All the patients who were alive had grade III tumors, while no survivors were observed among patients with grade IV tumors. Specifically, patients with anaplastic oligodendroglioma had a higher survival rate (58.8% alive n=10) compared to those with GBM (0% alive). The median OS for the entire cohort was 15 months, with significant differences observed between tumor grades.

Prognostic factors

Several prognostic factors were associated with improved survival outcomes (Table 2). Females constituted 45.0% (n=8) of survivors, suggesting a trend toward better survival in females, though this difference was not statistically significant. Grade III and oligodendroglioma histology were associated with better survival. The most common adverse events included hematologic toxicity, fatigue, and neurocognitive decline. However, these toxicities did not affect survival.

**Table 2:** Prognostic factors The Mann-Whitney test used for statistical analysis

Prognostic factors		Total patients (%)	Alive (%)	p-Value
Gender	Male	47 (65.3)	10 (21.2)	0.035
	Female	25 (34.7)	8 (32)	
Histology	Glioblastoma	40 (55.5)	0 (0)	0.004
	Astrocytoma	17 (23.6)	8 (47)	
	Oligodendroglioma	15 (20.8)	10 (66.6)	
Grade	Grade III	28 (38.8)	18 (64.2)	0.001
	Grade IV	44 (61.1)	0 (0)	

Disease progression and recurrence

Disease progression was strongly associated with mortality, as 79% (n=43) of deceased patients had documented progression compared to only 5.5% of survivors. Recurrence was observed in two survivors and four deceased patients. Median PFS was eight months.

## Discussion

HGG is usually treated as an incurable disease. In order to improve the oncological outcomes for this condition, numerous investigations have been conducted. The EORTC (European Organisation for Research and Treatment of Cancer) conducted a randomized phase III clinical trial that established the current standard of care for glioblastoma. This study compared adjuvant radiotherapy (RT) alone to concurrent chemoradiotherapy (CTRT) with TMZ, followed by adjuvant TMZ for six cycles in newly diagnosed GBM undergoing maximal safe resection. The trial demonstrated that the combined treatment approach significantly improved both median and 2-year survival rates compared to RT alone. [12]. Of the 573 GBM patients in the aforementioned trial, 82% underwent surgery, 85% finished the concurrent plan, and only 47% finished the adjuvant cycles. Compared to RT alone, the RCT's HR of mortality was 0.63 (95% CI: 0.52-0.75; p = 0.001). Compared to 10.9% (95% CI: 7.6%-14.8%) with RT alone, the two-year survival rate with combination therapy was 27.2% (95% CI: 22.2%-32.5%). The OS was 9.8% at five years and 1.9% at 10 years. PFS was 6.9 months [(5.8-8.2) with 95% CI[ as opposed to 5 months [(4.2-5.5) with 95% CI]. Only 5% of patients stopped treatment despite the fact that 16% of patients experienced significant myelosuppression as one of the side effects [7].

The goal of this study was to analyze OS in patients with HGGs who underwent surgery, adjuvant chemoradiotherapy, and adjuvant TMZ therapy. The results emphasize the difficulties in treating these aggressive brain tumors while also highlighting the crucial roles that tumor grade, histological subtype, and length of treatment play in determining survival outcomes. The median age of our cohort was 63 years (IQR 54.5-70.3), which is fairly similar to the characteristics documented in previous research [13]. In Nunna et al.’s study [14], the proportion of patients over 65 was 45.8%; in the current report, it was 42.9%.

In our study, the median OS was 15 months. Our findings are similar to those reported in previous research. Patients with GBM had a poor median OS of around 9-13 months, while those with oligodendroglioma had an OS of around 16-19 months [7]. Nunna et al. [14] analyzed 104,456 GBM patients from 2004 to 2016 by analyzing the National Cancer Database (NCDB) and found that the five-year OS was 5.3% and the mean OS was 9.1 months. Using 3,895 GBM patients, Shieh et al. [15] achieved a median survival of 12.6 months. Kang et al. [16] reported that at one, two, and five years, relative survival rates for 5,754 GBM patients were 12.1%, 30.4%, and 59.3%, respectively. Hansen et al. [17] reported that the median OS for all GBM patients was 11.2 months. In contrast, Fabbro-Peray et al. [18] reported a median OS of 25.5 months in patients who underwent surgery and received adjuvant Stupp regimen (six cycles of TMZ). According to Kuo et al. [19], the mean OS time in 2,379 patients with HGGs who received adjuvant TMZ was 50.3 months.

Significant differences in survival according to tumor grade were also found in the study; the worst prognosis was linked to grade IV tumors (GBM). While patients with grade III tumors showed higher survival rates, none of the GBM patients were alive. This is consistent with the body of research that shows that HGGs are linked to worse outcomes because of their aggressive biological behavior, which includes resistance to therapy, fast proliferation, and invasiveness [20,21]. The lack of survivors among GBM patients emphasizes the need for more potent treatment approaches for this patient population.

This study found a number of prognostic markers, such as treatment mode, tumor grade, and histological subtype. Given that oligodendroglial tumors are known to have a favorable prognosis because of their unique molecular profile, which includes IDH mutations and 1p/19q co-deletion, patients with anaplastic oligodendroglioma showed a greater survival rate than those with GBM [22]. There was also a tendency for females to have higher survival rates, albeit this difference was not statistically significant. Although the underlying mechanisms are still poorly understood, this discovery is consistent with some research studies that imply gender-based differences in glioma biology and outcomes [23].

Mortality was closely linked to the course of the disease; fewer survivors had documented progression than the majority of deceased individuals. This emphasizes how crucial it is to attain disease control by the most safe resection possible and efficient adjuvant treatment. The difficulties presented by the infiltrative character of HGGs and their tendency for local recurrence were further highlighted by the observation of recurrence in 10.0% of survivors and 16.3% of patients who passed away [24].

There were no appreciable variations in the toxicity profiles of the various treatment regimens, and adjuvant therapy was typically well tolerated. Hematologic toxicity, tiredness, and neurocognitive deterioration were the most frequent adverse events, which is in accordance with the recognized side effects of radiation and TMZ [25]. These results emphasize how crucial it is to keep an eye on and control treatment-related toxicities in order to preserve patients' quality of life while undergoing therapy.

The growing use of immunotherapy offers a path for its conjunction with tumor-treating fields (TTFields) in the treatment of GBM. In patients with newly diagnosed GBM, a phase 1 trial (NCT03223103) will evaluate the safety and tolerability of TTFields in conjunction with a mutation-derived tumor antigen vaccination during the maintenance phase of TMZ therapy. NCT03405792 (2-THE-TOP), another phase 2 trial, examines if pembrolizumab, an anti-PD-1 monoclonal antibody, improves the immune responses triggered by TTFields in newly diagnosed GBM patients. In comparison to matched control patients in the EF-14 study, who had an OS of 15.9 months and a PFS of 7.9 months, respectively, the early results revealed an increased OS of 25.2 months and a PFS of 12.1 months [26,27].

The retrospective form of this study is one of its many drawbacks, as it may add selection bias and restrict how broadly the results can be applied. The statistical power of the analysis may also be impacted by the comparatively small sample size, especially for specific histological subtypes. Furthermore, molecular markers that are known to have a substantial impact on treatment response and survival outcomes, such as IDH mutations or MGMT promoter methylation status, were not taken into consideration in this investigation [28,29]. Molecular profiling should be used in future research to give a more thorough picture of the variables affecting survival in HGG patients. Another limitation of the study is the small sample size. As the sample size was small in our study, and the follow-up period was less, larger prospective randomized studies with a longer duration of follow-up are needed for a strong evaluation of survival.

## Conclusions

Despite ongoing research and studies, the OS rate for patients with GBM is still low. OS has improved with maximum safe resection, followed by adjuvant concurrent CTRT and adjuvant TMZ. The OS benefit was higher for female patients, grade III, astrocytoma, and oligodendroglioma histologies. This report emphasizes how aggressive HGGs, especially GBM, can be and how difficult it is to achieve long-term survival using the existing treatment approaches.
